# Considerations in Premature Menopause: A Review

**DOI:** 10.7759/cureus.69744

**Published:** 2024-09-19

**Authors:** Apoorva Dave, Dharmesh J Patel, Deepti Shrivastava, Kamlesh Chaudhari, Rahul Manchanda

**Affiliations:** 1 Department of Obstetrics and Gynecology, Jawaharlal Nehru Medical College, Datta Meghe Institute of Higher Education and Research, Wardha, IND; 2 Department of Obstetrics and Gynecology, Holy Family Hospital, New Delhi, IND

**Keywords:** bilateral oophorectomy, estrogen, induced menopause, ovarian failure, premature menopause, spontaneous menopause

## Abstract

Premature menopause impacts 1% of women under the age of 40. The women are at risk of premature death, ischemic disease of the heart, neurological conditions, mood disturbances, psychosexual problems, osteoporosis, and subfertility. There is an imperative for less complicated protocols and enhanced approaches for oocyte donation to get pregnant and achieve motherhood in at-risk women.

A review of the pertinent literature on premature ovarian insufficiency and selected references was done. A comprehensive review was undertaken by searching the databases PubMed, Scopus, EMBASE, Web of Science, and Science Direct.

Pregnancy in women with premature menopause was formerly uncommon, but because of recent advances in oocyte donation, women with premature menopause can now aspire to have a child. Hormone replacement treatment is useful in treating the negative effects of premature ovarian insufficiency.

Women who experience early menopause are at risk for early mortality, ischemic heart disease, neurological conditions, mood problems, psychosexual disorder, osteoporosis, and subfertility. Public awareness and education are critical tools for saving women at peril.

## Introduction and background

A rare yet significant cause of sex hormone deficit and subfertility in premenopausal women is known as primary ovarian insufficiency (POI). Characteristics of POI include menopausal levels of the hormone follicle-stimulating hormone (FSH) and irregular or absent menstruation occurring before the age of 40. Women who exhibit these symptoms after age 40 but before reaching 45 are considered in the early menopausal stage, as the typical age of natural menopause falls between 48 and 52 years [1]. One in 100 women under the age of 40 and women below the age of 30 experience spontaneous POI. Five percent of women are said to have early menopause before the age of 45 [2]. Numerous medical conditions attributed to POI directly correlate with ovarian hormone deficiencies, particularly estrogen ones. This emphasizes the value of physiologic hormone replacement treatment for POI-affected women. Unfortunately, information on negative consequences from the Women's Health Initiative (WHI) trial, a study involving elderly postmenopausal females, has led many to advise against the use of estrogen-progestin therapy (EPT) or estrogen therapy in young females with POI or premature menopause [3]. According to the WHI, using EPT is associated with elevated health hazards, including a higher chance of developing breast carcinoma, stroke, and cardiac compromise [4]. This is noteworthy because young females with POI and premature menopause have a chronic level of estrogen deficiency compared to their peers with normally functioning ovaries. This is in contrast to women who have normal menopause. Hormone replacement therapy (HRT) is entirely accurate for women with POI and early menopause because the prescription hormones replace naturally produced hormones. The different facets of POI, including its definition, etiology, clinical presentation, diagnosis, and management, will be covered in this chapter. It will also examine the many methods for managing and preventing early ovarian insufficiency, emphasizing interventions to improve mental health and medical and lifestyle modifications [5,6].

## Review

Materials and methods

**Figure 1:**
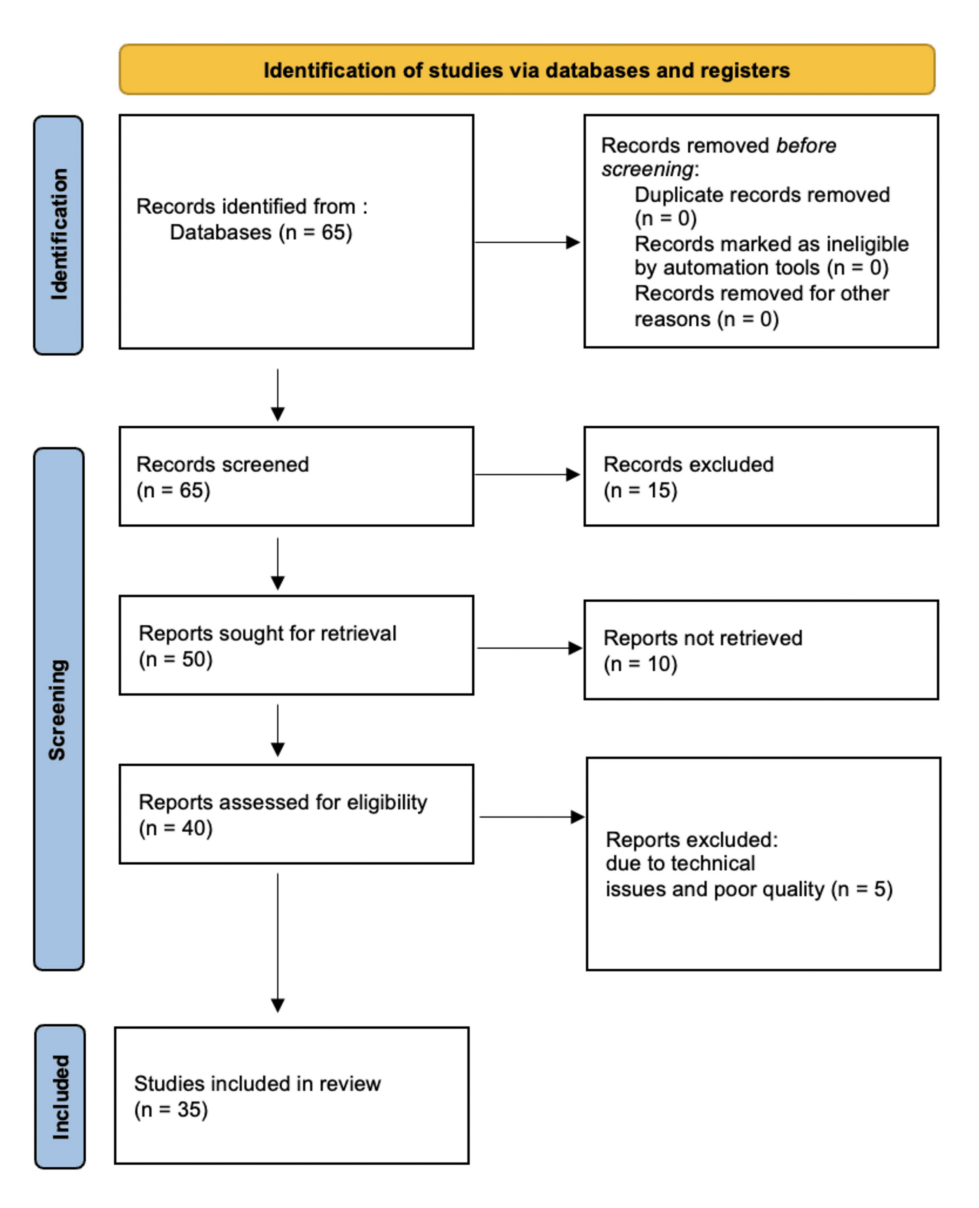
Preferred Reporting Items for Systematic Reviews and Meta-Analyses flow diagram for the selection of review materials

A comprehensive review was undertaken by searching PubMed, Scopus, EMBASE, Web of Science, and Science Direct databases. Various studies describing premature ovarian insufficiency were included in the present study. We searched for studies until November 2023. The search strategy included keywords such as premature menopause, premature ovarian insufficiency, treatment, and diagnostic strategies in such cases. A total of 65 articles were found, but only 35 were chosen to be included because it was determined that they were pertinent. These were selected following the Preferred Reporting Items for Systematic Reviews and Meta-Analyses guidelines. A comprehensive outline of the selection method is shown in Figure 1.

Etiology of premature menopause

The etiology of premature menopause is mentioned in Table 1.

**Table 1:** Etiology of premature menopause POI: primary ovarian insufficiency; PCOS: polycystic ovary syndrome; APS: autoimmune polyendocrine syndrome

Type of cause [7-10]	Examples
Genetic causes	Chromosomal defects: Turner syndrome or fragile X syndrome. Family history: there is a risk of potential genetic predisposition, so a family history of POI is important to elicit (14% cases)
Autoimmune factors	Autoimmune oophoritis (4% of primary ovarian insufficiency): in some cases, the immune system mistakenly attacks and damages the ovarian tissue, leading to ovarian dysfunction. This condition is known as autoimmune oophoritis
Iatrogenic	Radiotherapy and chemotherapy: pelvic radiation and chemotherapy for the treatment of cancer can affect the ovaries and cause POI. The extent of damage is dose-dependent. Surgical procedure: certain surgical procedures, such as the removal of ovarian cysts or the removal of one ovary, can reduce ovarian function and potentially lead to POI
Environmental factors	Toxins and chemicals: environmental toxins, like industrial chemicals, certain pesticides, and toxins from active and passive smoking, may contribute to POI and early menopause-like symptoms. Infections: few infections, like mumps, can lead to POI rare cases
Metabolic and hormonal disorders	PCOS: in a few cases of PCOS, women may attain premature menopause. Hypothyroidism: hypothyroidism may influence the average hormonal balance and potentially lead to ovarian dysfunction
Idiopathic reasons	In various cases when no known cause is present (approximately 90% of cases)
Mutations in the genes	Some genetic mutations like FMR1 (2%-5%) (linked with fragile X syndrome), FOXL2, and GDF9 are associated with POI
Autoimmune causes	APS is a rare autoimmune disorder that can affect multiple endocrine glands, including the ovaries, and contributes to ovarian dysfunction
Environmental factors/habits	Smoking: as tobacco in the cigarettes imparts toxic effects on the ovaries. Exposure to chemicals: when exposed for a long time, certain chemicals may lead to ovarian dysfunction

It is crucial to note that the actual cause of POI may differ within individuals. A thorough evaluation by the gynecologist, including hormone tests, genetic testing, and a medical history review, can help identify the underlying cause and guide appropriate management and treatment options. Early diagnosis and intervention are essential for addressing POI's symptoms and potential complications [10].

Clinical presentation

Symptoms

Women having POI may present with various symptoms, which can mimic natural menopause but occur at a very young age. It is essential to remember that every single woman's clinical presentation is unique, and some females with POI may not get any symptoms at all or have atypical presentations. Changes in menstrual patterns, such as irregular periods or secondary amenorrhea, combined with symptoms associated with estrogen insufficiency, such as hot flashes, night sweats, mood swings, and vaginal dryness, are usually the most prevalent clinical features. Early recognition and assessment by the gynecologist are essential for proper management and meeting the distinctive needs of women with POI [9,10]. Nelson found that amenorrhea was most common, followed by hot flashes and night sweats. Additionally, a significant family history of similar symptoms was present in most women [11].

Complications

POI may significantly and extensively impact a woman's health and quality of life. Possible long-term effects include an increased risk of osteoporosis, psychological discomfort, and cardiovascular disease [12]. Addressing these complications is essential to managing POI. The following are a few POI-related complications.

Osteoporosis and Fracture Risk

Women with POI are more susceptible to osteoporosis (decreased bone mineral density) and fractures, mainly if they are not on HRT. Fractures are more likely in POI because of reduced bone density brought on by estrogen decline [12,13]. Albright et al. are the first to show a link between estrogen insufficiency, menopause, and a greater likelihood of fractures in women [14].

Cardiovascular Disease

There may be an increased risk of cardiovascular disease in females with POI, which may lead to increased mortality [15-17]. Estrogen has cardioprotective properties and can help avoid cardiovascular disease [18]. Hormonal alterations related to POI, specifically the reduction of estrogen, might impact lipid profiles and heighten the risk of cardiovascular events [18,19].

Impact on Psychological Well-Being

Psychological symptoms like depression, anxiety, and a decreased quality of life are common in women with POI. Emotional despair can result from the consequences of subfertility, hormonal changes, and adapting to the diagnosis [20].

Sexual Dysfunction

Vaginal dryness and reduced sexual desire are two symptoms of sexual dysfunction that women with POI are more prone to encounter. These problems can significantly impact a woman's relationships and sexual health [21,22].

Subfertility

One of the primary consequences of POI is infertility. While some women may have choices with fertility preservation techniques like oocyte cryopreservation, they have poor success rates [23]. However, few studies have shown positive pregnancy outcomes in such cases [24,25].

Cognitive Function

Studies have looked into how POI-related hormonal changes could affect cognitive performance. Women with POI might notice mild changes in memory and attention, two areas of cognitive function [25].

Vasomotor Symptoms and Quality of Life

Women with POI frequently have vasomotor symptoms, including night sweats and hot flashes, which can negatively affect their quality of life [26-28].

Diagnosis

POI can be predicted in the following females [26,27]: those who are more than 35 years of age, those whose biological mother had POI, those who suffer from an autoimmune disorder or have genetic reasons, and those with a history of pelvic surgery, chemotherapy, or radiotherapy.

Diagnostic Criteria

The diagnosis is typically confirmed when a person experiences oligo/amenorrhea for at least four months, along with increased FSH levels of more than 25 international units (IU)/L on two separate occasions with a gap of at least four weeks between the tests. This diagnosis also applies to women under 40 who experience irregular menstruation or amenorrhea for at least four months and have low estrogen levels [28].

Tests to Conduct

To identify the root cause of POI and rule out other conditions with comparable symptoms, genetic testing is performed in addition to a comprehensive medical history, physical examination, and hormone testing [28]. Chromosomal analysis is done in all the women after iatrogenic causes of POI have been ruled out. Fragile X premutation testing discusses the implications of this entity before conducting this test. A screening test for 21 hydroxylase antibodies, also known as adrenocortical antibodies, was done. If this test result is positive, the patient must be referred to an endocrinologist to test for adrenal function and exclude Addison's disease. If an immune disorder or unknown etiology is suspected, a thyroid antibody is found, and TPO Ab is checked. If karyotyping comes positive, the woman must be referred to an endocrinologist, cardiologist, and geneticist. However, if there is a high index of clinical suspicion in a negative test, the next karyotyping test should be conducted in epithelial cells. The presence of the Y chromosome warrants gonadectomy, so it should be discussed with the patient [29]. If the TPO antibody test comes positive, TSH must be checked every year, and if the test comes negative with positive signs and symptoms, retesting is done [30].

Management of POI

The goals of the treatment in the cases of POI are to replenish hormones that the ovaries no longer produce, to manage the symptoms or consequences of POI (like night sweats, vaginal dryness, etc.), to lessen the risk of conditions that may arise due to POI, and to manage the underlying health problems that increase POI symptoms.

Lifestyle Changes

A healthy lifestyle that incorporates regular exercise, a balanced diet, and stress management can help reduce symptoms of POI and enhance general well-being.

Diet Modifications

A calcium- and vitamin D-rich diet is essential for preserving bone health in women with POI. Furthermore, maintaining a healthy body weight and regular exercise will improve general well-being and help control some of the symptoms associated with POI. The recommended daily allowance of vitamin D3 (cholecalciferol) and elemental calcium in women with POI is 103 to 2 × 103 IU and 12,00 mg, respectively [29].

Stress Management

Premature menopause can disrupt a woman's sense of identity and femininity, leading to feelings of loss, grief, and low self-esteem. Psychosocial support, including counseling and support groups, can help women navigate these challenges and develop coping strategies. Stress can aggravate POI symptoms and have a detrimental impact on a woman's overall well-being. Mindfulness, yoga, meditation, and relaxation methods can assist women with POI in managing stress and enhancing their quality of life [29].

Hormone Replacement Therapy

POI treatment relies mainly on HRT. It is beneficial to offer estrogen replacement medication to women experiencing POI [29-31]. Its goal is to restore and replenish the deficient hormones (estrogen and progesterone) to ease symptoms and lower the risk of consequences such as osteoporosis. Physiologic HRT (transdermal estradiol 100-150 mcg/day and cyclic progesterone) is more successful than continuous combined therapy with oral contraceptives in preserving bone health in young women having POI. There is growing evidence that restoring normal estrogen levels can be preventive against cardiovascular disease, osteoporosis, and potentially dementia [32]. As a result, many people contemplate using HRT for a short period [32-35]. However, HRT must be individualized and carefully examined for possible hazards and contraindications. HRT manages symptoms such as hot flashes, vaginal dryness, and mood swings. It may also help to preserve bone density and lower the risk of cardiovascular disease caused by estrogen shortage. However, HRT may not be appropriate for all women, particularly those with a history of certain medical disorders such as breast cancer. Individualization requires the healthcare professional to weigh the advantages and risks of HRT meticulously.

Fertility Preservation

Women with POI who want to preserve their fertility can choose oocyte cryopreservation (egg freezing) or ovarian tissue cryopreservation. Such procedures enable women to save their eggs or ovarian tissue for future use through IVF or transplantation. While these approaches provide hope for future conceptions, success rates vary, and it is critical to discuss with a fertility expert before pursuing these choices.

Bone Health Management

Estrogen plays a significant role in maintaining bone density. Therefore, women with POI are at increased risk of osteoporosis. Calcium and vitamin D supplements, weight-bearing exercises, and bone density monitoring through dual-energy X-ray absorptiometry scans are some measures to help manage bone health [29].

Psychological Support

Counseling and support groups may be highly beneficial in dealing with the diagnosis, stress management, and handling body image and self-esteem issues.

Support Groups and Counseling

A POI diagnosis can be emotionally upsetting since it can affect a woman's self-esteem, body image, and sense of womanhood. Women can manage the emotional issues connected with POI with psychological support, such as counseling or attending support groups. These support networks provide a secure area for people to share their experiences, acquire coping methods, and build an aura of community [32].

Education and Information

It is critical to provide women with information regarding their health. Understanding the cause, symptoms, and treatment choices available can assist women in making well-informed choices and reduce anxiety. Gynecologists should provide educational tools and participate in open conversations with their patients to answer questions and concerns.

Considering the long-term health sequelae of POI, the focus must be on decreasing its incidence [34]. Modifiable factors, including modifications in gynecological surgical practice, altering the treatment regimens to treat malignant and chronic diseases, and genetic counseling, must not be ignored to lessen its burden.

## Conclusions

For women with POI or early menopause, HRT is crucial for managing symptoms, preserving bone density, and reducing risks of cardiovascular disease and dementia. HRT should mimic natural ovarian hormone production, with cyclical progestin to protect the endometrium. Postmenopausal adjustments or discontinuation of HRT should be based on individual needs and risks. Regular annual reviews are essential for those on HRT. Transdermal estradiol is preferred over oral forms for more physiological hormone levels. Future research should aim to understand the causes of premature menopause, develop early detection biomarkers, and create targeted therapies to maintain ovarian function and assess long-term health outcomes.
